# Supplementary material for: The role of feedforward and feedback inhibition in modulating theta-gamma cross-frequency interactions in neural circuits
Source: PLoS Comput Biol. 2025 Aug 13;21(8):e1013363. doi: 10.1371/journal.pcbi.1013363 (PMC12393765; doi:10.1371/journal.pcbi.1013363)
Supplement: S1 Table — Synaptic weights for Fig 1. The synaptic weight depicted for PC → BC is for the θ-PING, otherwise it is zero. Similarly, the synaptic weight depicted for θ → BC is for the θ-ING, otherwise it is zero. The difference in the order of magnitude between PC → BC and θ → BC is due to differences in the number of presynaptic neurons (80 vs 500) and their activity (0.49Hz vs 8Hz). (PDF) [file pcbi.1013363.s001.pdf]

Connection	Post. Site	Receptor	g(nS)	#pres.
θ→PC	Distal	AMPA	0.04	500
		NMDA	0.004	500
BC→PC	soma	$GABA_A$	0.14	30
Noise→PC	soma	AMPA	0.22	1
Noise→PC	soma	$GABA_A$	0.24	1
Pois.→PC	Proximal	AMPA	0.01	1
		NMDA	0.001	1
BC→BC	soma	$GABA_A$	8.1	30
Noise→BC	soma	AMPA	0.08	1
Noise→BC	soma	$GABA_A$	2	1
PC→BC	soma	AMPA	40	80
		NMDA	4	80
θ→BC	soma	AMPA	0.5	500
		NMDA	0.05	500
