# Supplementary material for: The role of feedforward and feedback inhibition in modulating theta-gamma cross-frequency interactions in neural circuits
Source: PLoS Comput Biol. 2025 Aug 13;21(8):e1013363. doi: 10.1371/journal.pcbi.1013363 (PMC12393765; doi:10.1371/journal.pcbi.1013363)
Supplement: S2 Table — NMDA has an additional scaling factor due to the magnesium block 1/(1+0.28[Mg]e−0.062V), where [Mg] = 1mM is the concentration of magnesium, and V the membrane potential in mV. (PDF) [file pcbi.1013363.s002.pdf]

Receptor	τ_r(ms)	τ_d(ms)
AMPA	0.05	5.3
NMDA	15	150
$GABA_A$	0.07	9.10
