# Supplementary material for: The role of feedforward and feedback inhibition in modulating theta-gamma cross-frequency interactions in neural circuits
Source: PLoS Comput Biol. 2025 Aug 13;21(8):e1013363. doi: 10.1371/journal.pcbi.1013363 (PMC12393765; doi:10.1371/journal.pcbi.1013363)
Supplement: S3 Table — All other synaptic parameters are as in θ-ING (see S1 Table). (PDF) [file pcbi.1013363.s003.pdf]

Conn.	w_i (nS)	w_{ii}(nS)	w_{iii}(nS)	w_{iv}(nS)
BC→PC	0.0	0.14	0.29	0.43
θ→PC	0.0	0.03	0.05	0.06
θ→BC	0.3	0.4	0.5	0.6
