# Supplementary material for: The role of feedforward and feedback inhibition in modulating theta-gamma cross-frequency interactions in neural circuits
Source: PLoS Comput Biol. 2025 Aug 13;21(8):e1013363. doi: 10.1371/journal.pcbi.1013363 (PMC12393765; doi:10.1371/journal.pcbi.1013363)
Supplement: S4 Table — The simulations are the same as in Fig 3. Blue-colored rates are associated with motifs that exhibit negative CFD. (PDF) [file pcbi.1013363.s004.pdf]

	$f_{r,BC}$(Hz)				$f_{r,PC}$(Hz)			
Conn.	w_i	w_{ii}	w_{iii}	w_{iv}	w_i	w_{ii}	w_{iii}	w_{iv}
BC→PC	11.81	11.81	11.81	11.81	1.85	0.48	0.13	0.06
θ→PC	11.81	11.81	11.81	11.81	0	0.22	1.31	6.39
θ→BC	5.62	8.71	14.71	17.18	0.95	0.67	0.34	0.27
