# Supplementary material for: The role of feedforward and feedback inhibition in modulating theta-gamma cross-frequency interactions in neural circuits
Source: PLoS Comput Biol. 2025 Aug 13;21(8):e1013363. doi: 10.1371/journal.pcbi.1013363 (PMC12393765; doi:10.1371/journal.pcbi.1013363)
Supplement: S5 Table — As also stated in S1 Table, the difference in the order of magnitude between PC → BC and θ → BC is due to differences in the number of presynaptic neurons (80 vs 500) and their activity (0.49Hz vs 8Hz). (PDF) [file pcbi.1013363.s005.pdf]

Conn.	w_i (nS)	w_{ii} (nS)	w_{iii} (nS)
PC→BC	0	20	40
θ→BC	0.0	0.25	0.5
