# Supplementary material for: The role of feedforward and feedback inhibition in modulating theta-gamma cross-frequency interactions in neural circuits
Source: PLoS Comput Biol. 2025 Aug 13;21(8):e1013363. doi: 10.1371/journal.pcbi.1013363 (PMC12393765; doi:10.1371/journal.pcbi.1013363)
Supplement: S6 Table — (PDF) [file pcbi.1013363.s006.pdf]

Conn.	θ-ING	C1	C2	θ-PING
PC→BC	0	10	20	60
θ→BC	0.5	0.42	0.33	0.0
