# Supplementary figures and images for: The role of feedforward and feedback inhibition in modulating theta-gamma cross-frequency interactions in neural circuits
Source: PLoS Comput Biol. 2025 Aug 13;21(8):e1013363. doi: 10.1371/journal.pcbi.1013363 (PMC12393765; doi:10.1371/journal.pcbi.1013363)

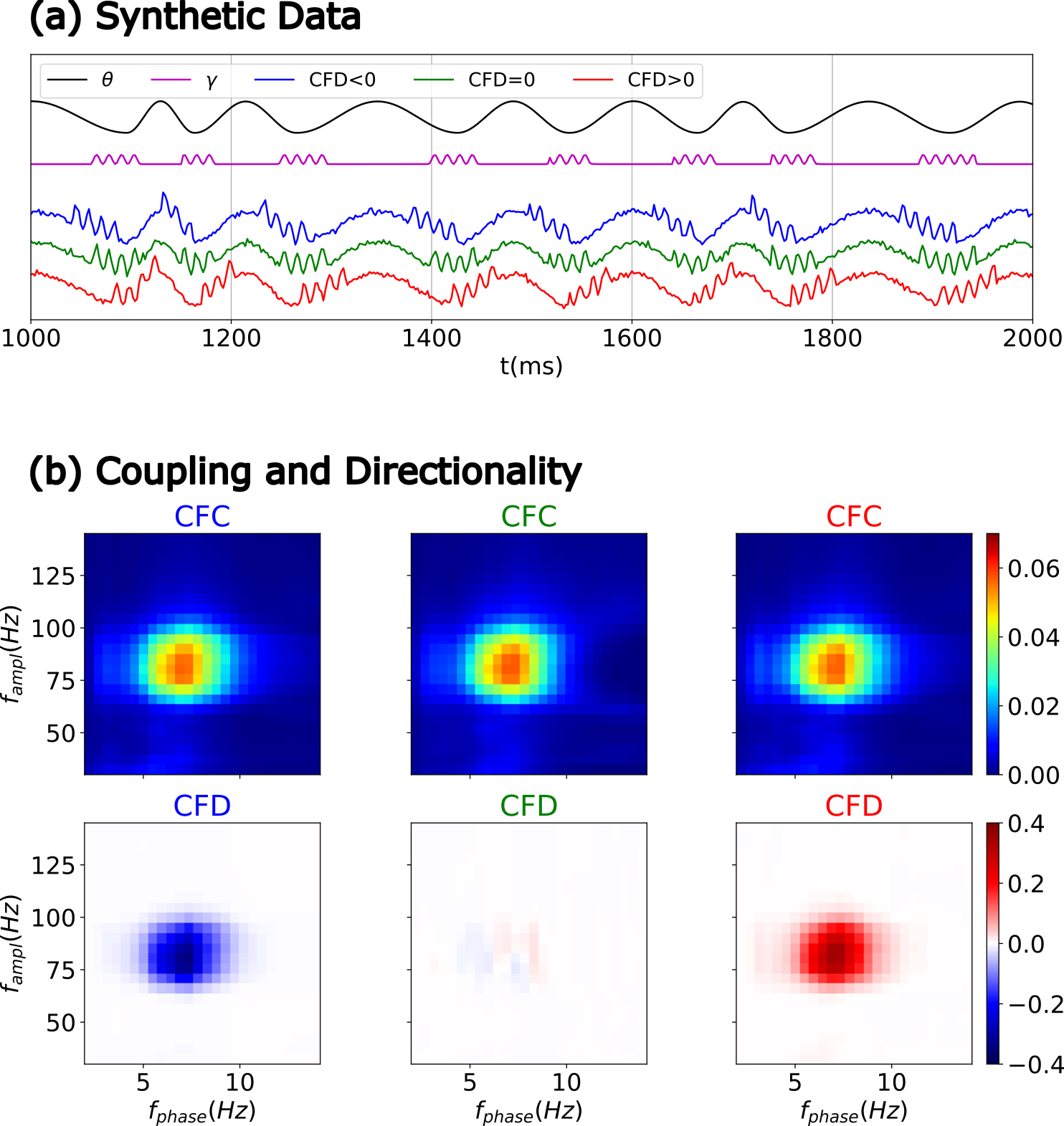

Supplement: S1 Fig — (a) Synthetic data of different CFD made following [29]. The θ signal was generated by concatenating sinusoidal segments of mean amplitude 10 and different periods drawn from a Gaussian of mean 125ms. The γ signal is an 80Hz sinuisoidal coupled to the trough of θ. Then, θ and γ are added together to create 3 timeseries with different theta-gamma relationship: in the blue line γ is lagged 10ms behind θ, in the green timeseries γ is not lagged whereas for the red line θ laggs behind γ by 10 ms. (b) Top: Cross Frequency Coupling of the timeseries in (a). Notice that independently of the time difference between theta and gamma all timeseries have identical CFC peaking at (8Hz,80Hz). (c) Bottom: Cross Frequency Directionality of the lines in (a). CFD is able to detect the time delay between the γ amplitude and θ phase. (TIFF) [file pcbi.1013363.s007.tiff]

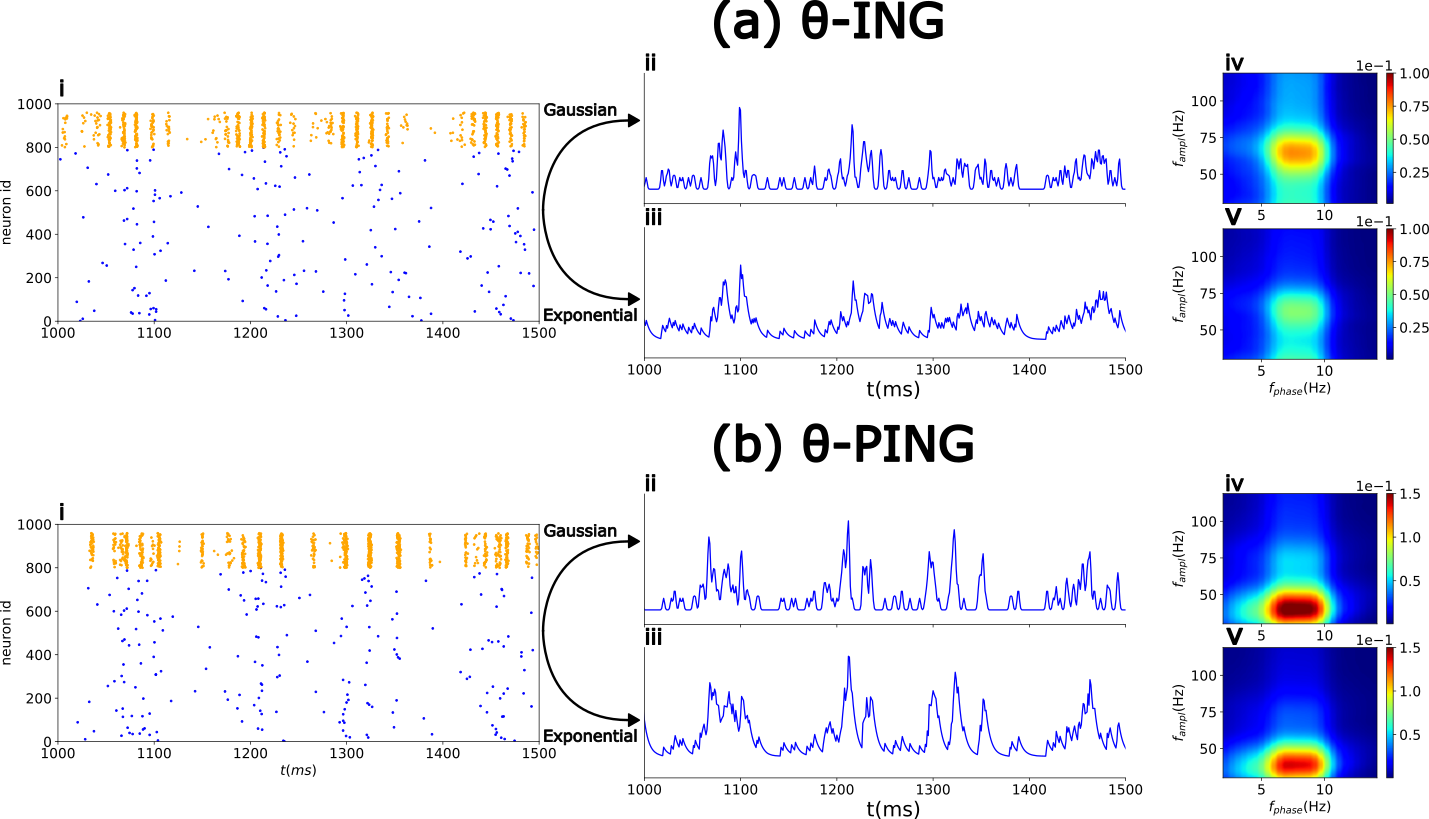

Supplement: S2 Fig — (i) Raster plots of PCs in blue (and BCs in orange for completeness). The convolution of each PC spike with either a 1 ms Gaussian kernel (ii) or a 5 ms decaying exponential kernel (iii) generates a proxy for the instantaneous output firing rate (in arbitrary units). A CFC analysis of the firing rate (iv, v) provides insight into what could be detected in the dendrites of a downstream layer which is not dependent on the kernel. This analysis was conducted for PC spikes in a θ-ING motif (a) and a θ-PING motif (b). To ensure sufficient spikes for the CFC analysis, both networks were scaled up by a factor of 4—achieved by increasing the number of neurons and projections while reducing the number of synaptic weights. (TIFF) [file pcbi.1013363.s008.tiff]

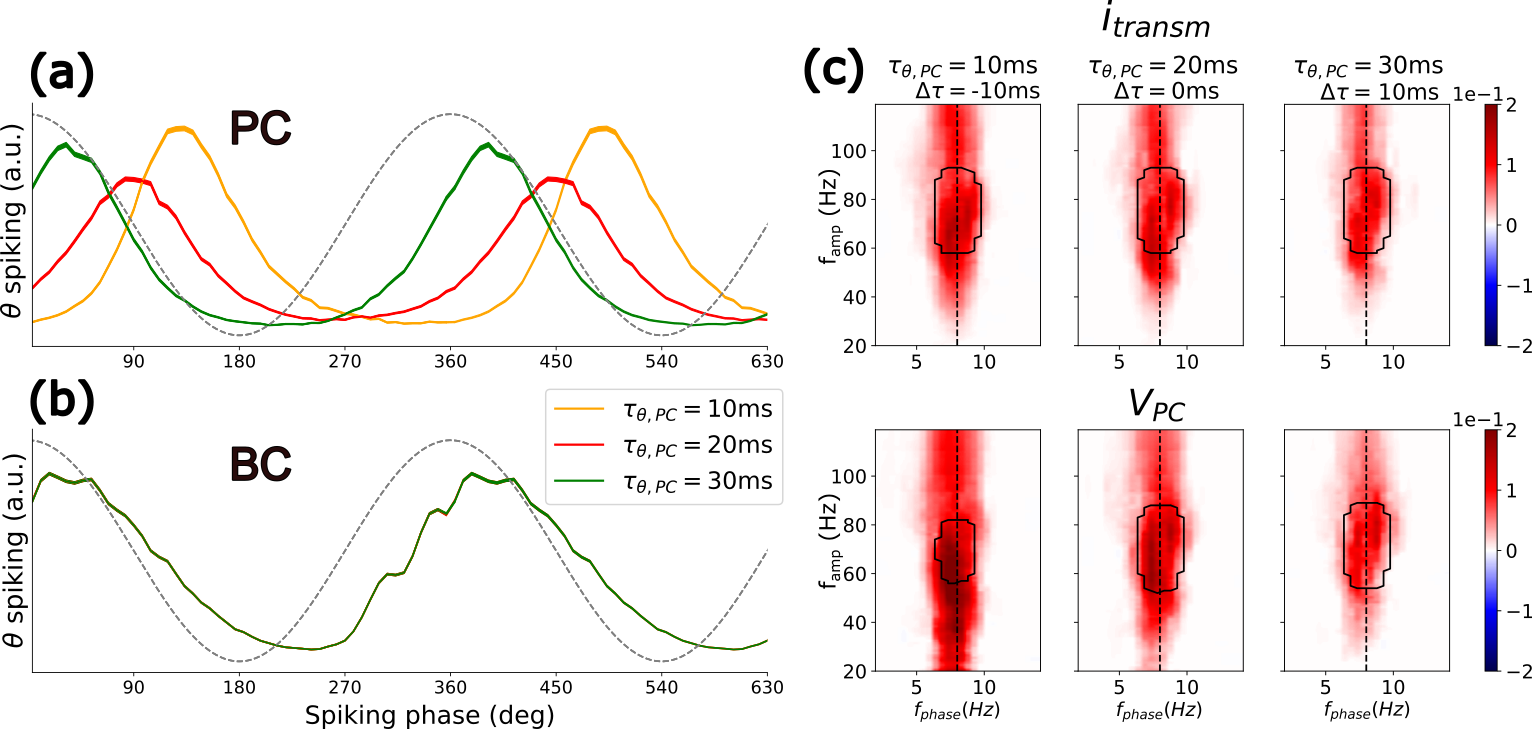

Supplement: S3 Fig — Panels (a) and (b) show the θ phase of PC and BC spiking, respectively. Panel (c) illustrates the CFD for itransm (top) and VPC (bottom). To derive the θ phase of the external population, spikes are passed through a decaying exponential kernel with a 5 ms time constant. Note that when using the external population’s θ phase, the BC phase remains unchanged, while the PC phase shifts significantly due to different offsets. Finally, as the local γ is consistently generated by the external θ driver, the CFD remains positive under all conditions. (TIFF) [file pcbi.1013363.s009.tiff]

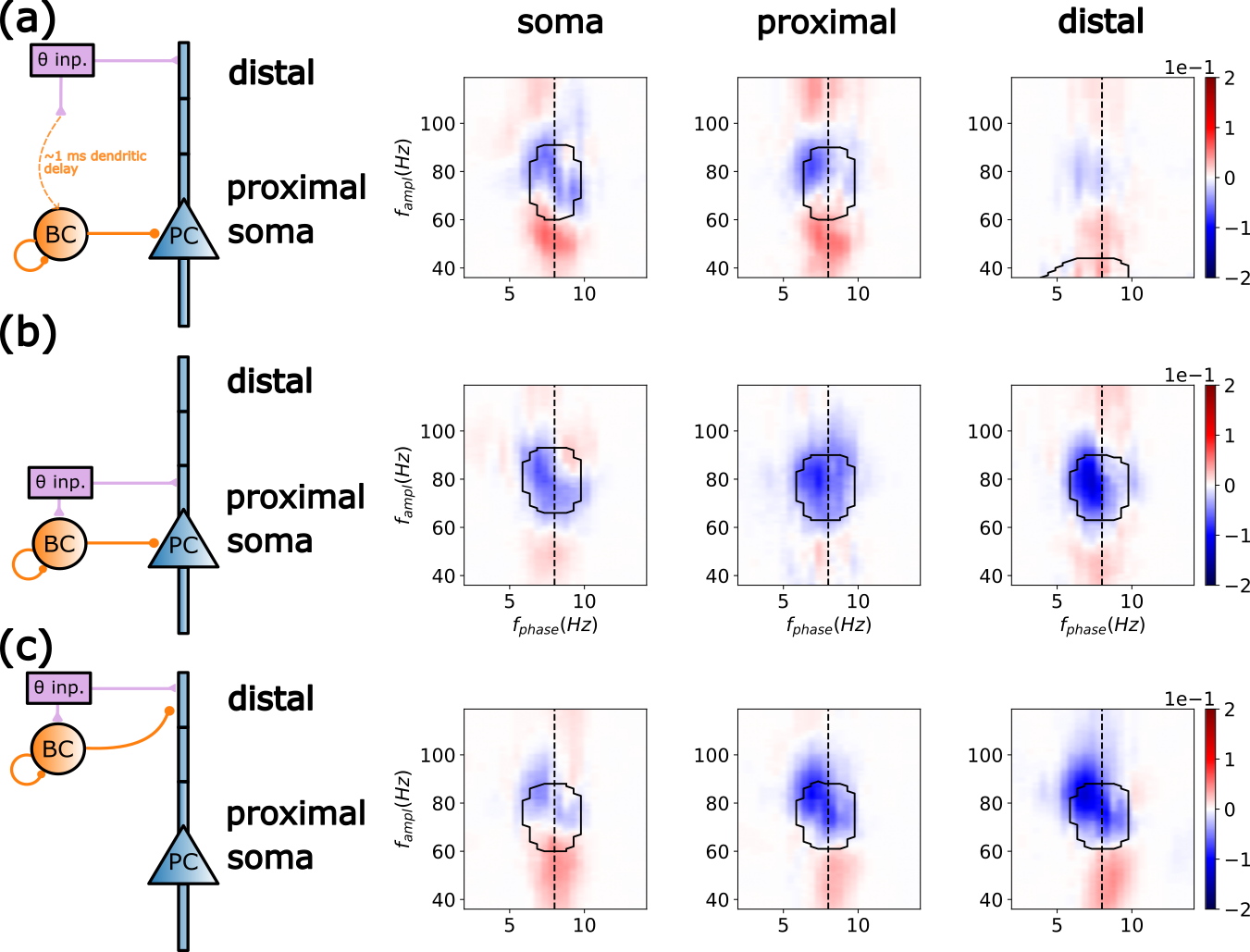

Supplement: S4 Fig — (a) The θ-ING motif analyzed in this study is visualized for reference (same as in Fig 1b-v) with the additional CFD of the transmembrane currents at the proximal and distal dendrites. (b) The theta input excites the BC and PC population closer to their somata. (c) The inhibitory population is positioned and projects at the same layer as the theta input. (TIFF) [file pcbi.1013363.s010.tiff]

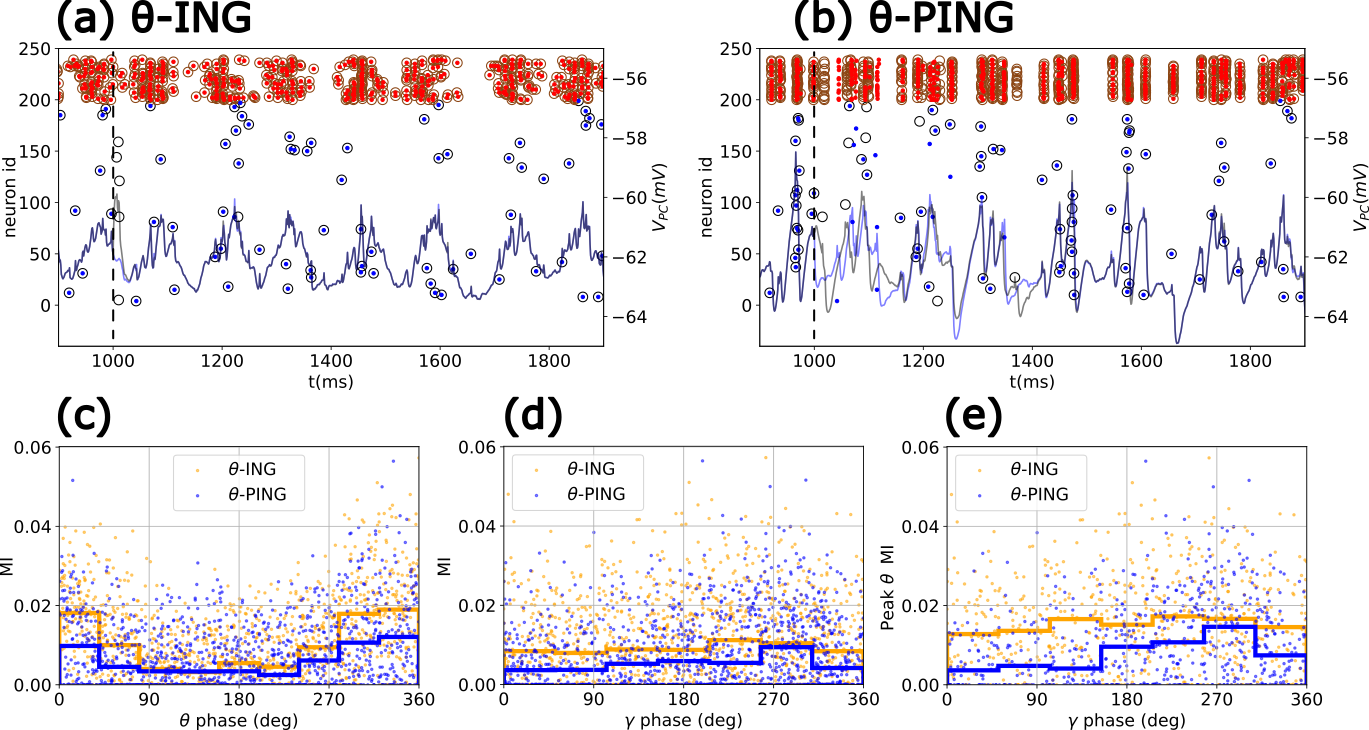

Supplement: S5 Fig — Single perturbation MI analysis. A single spike is introduced in the proximal dendrite at a predefined time within the interval (1-1.5) s (here at 1 s, indicated by the black dashed line). Panels (a) and (b) show the dynamics for a θ-ING and a θ-PING motif, respectively, both with similar firing rates. Blue lines represent the mean membrane potential at the PC soma (VPC,g) for the unperturbed case, while gray lines show the evolution after the perturbation (VPC,p). Open circles indicate spikes in the perturbed simulations (gray for PCs, brown for BCs), and solid circles represent spikes in the baseline condition (blue for PCs, red for BCs). Since only one perturbation is applied, the resulting encoding value between output and perturbation can be related to the network state at the time of perturbation. (c) Encoding values are plotted against the θ phase of VPC,p, with their histogram overlaid, where 180° represents the trough and 0°/360° the peak. (d) Same as (c), using a high-pass filter cutting of frequencies lower than 20 Hz to capture the gamma activity of both motifs. (e) Same as (d), but for encoding values only when the θ phase is between -90° and 90°, i.e., when the network is more depolarized by the θ input. In both panels (e) and (d) θ-PING MI depends on the γ phase more than θ-ING. (TIFF) [file pcbi.1013363.s011.tiff]
