# Supplementary material for: The role of feedforward and feedback inhibition in modulating theta-gamma cross-frequency interactions in neural circuits
Source: PLoS Comput Biol. 2025 Aug 13;21(8):e1013363. doi: 10.1371/journal.pcbi.1013363 (PMC12393765; doi:10.1371/journal.pcbi.1013363)
Supplement: Attachment — Submitted filename: Response Letter.pdf [file pcbi.1013363.s013.pdf]

Claudio R. Mirasso
Catedrático de Universidad

T. +34 971 172783
F. +34 971 173248
claudio@ifisc.uib-csic.es
http://ifisc.uib-csic.es/claudio

Palma de Mallorca, July 2nd, 2025.

Dr. Roman Bauer
Academic Editor
PLOS Computational Biology

Dear Dr. Bauer,

Please find below our point-by-point response to the reviewers' comments on the manuscript entitled "The Role of Feedforward and Feedback Inhibition in Modulating Theta–Gamma Cross-Frequency Interactions in Neural Circuits," by Dimitrios Chalkiadakis, Jaime Sánchez-Claros, Víctor J. López-Madrona, Santiago Canals and Claudio R. Mirasso. We hereby resubmit the manuscript in two formats—a clean copy and a version showing tracked changes—for your consideration in **PLOS Computational Biology**.

We gratefully thank the reviewers for their positive assessments and the many constructive suggestions that have strengthened our manuscript.

Sincerely,

Claudio R. Mirasso
on behalf of all authors

Reviewers' comments and Reviewer's Responses to Questions

Reviewer #1: Chalkiadakis et al. provide insights into the directionality of theta-gamma interactions in the hippocampus, focusing on the perspective of principal cells (PCs). They show that feedforward inhibition biases PC output toward gamma-to-theta directionality, whereas feedback inhibition induces the opposite, theta-to-gamma directionality. In a more realistic motif that integrates both ING and PING elements, they demonstrate that varying the relative strength of these components produces a continuum of gamma frequencies and theta-gamma directionalities. Their interpretation links these outputs to the interaction between theta inputs, basket cell activity, and PC responses. I find this an interesting theoretical contribution, but I have several conceptual comments regarding the manuscript, which I detail below.

Response: *We thank the reviewer for this positive global assessment.*

[a] In the experimental results, CFD analyses focus on the LFP component associated with EC inputs to CA1 (stratum lacunosum-moleculare). However, López-Madrona et al. (2020) also applied ICA to isolate pathway-specific components corresponding to CA3 inputs via the Schaffer collaterals (stratum radiatum). Given that CA3 is arguably the dominant excitatory drive to CA1, could the authors clarify why the EC-associated component was prioritized for the analyses shown in Figure 1? This question becomes particularly relevant given the authors' interpretation favoring an ING motif. While there is strong anatomical and functional evidence that CA3 inputs provide direct excitatory drive to CA1 PV-positive interneurons, I am not aware of comparably strong evidence for substantial direct excitation of these cells by EC inputs.

Related to this, the authors write in the discussion (lines 530–533): "Third, both in vivo and in vitro patch-clamp recordings from the CA1 region have demonstrated faster action potential initiation in BCs compared to PCs following stimulation of pathways that simultaneously excite BCs and PCs, namely the perforant path and the Schaffer collateral, respectively [48, 49]." While I agree that these papers demonstrate earlier BC spiking in response to Schaffer collateral stimulation, they do not appear to examine responses to perforant path input. As such, mentioning the perforant path hinting EC inputs to CA1 basket cells may be misleading. Indeed, basket cell dendrites typically do not extend into stratum lacunosum-moleculare. Although the alvear pathway might in principle mediate EC excitation of these interneurons, any such mechanism would require appropriate citation or further elaboration.

In sum, if a theta-modulated input is responsible for driving ING-like gamma in CA1, the CA3 pathway appears to be a more plausible candidate than EC. I may be overlooking a key aspect, and would welcome clarification or correction. Alternatively, if the EC component was selected for technical or interpretative reasons, it would be helpful for the authors to discuss this caveat and/or report CFD results based on the CA3-associated component as well.

Response: *We thank the reviewer for this thoughtful and detailed comment. We fully agree that CA3 represents a major excitatory input to CA1 and plays an important role in driving local circuit dynamics. However, our decision to focus on the EC-associated component in Figure 1 was based on both technical and conceptual considerations.*

First, experimental studies have shown that the strongest theta power in CA1 originates from entorhinal cortex (EC) layer III inputs; silencing CA3 has only a minor effect on CA1 theta power (see Fig. 1e of [12] and Fig. 2 of [64]; references according to the new manuscript). Because the EC component provides the highest signal-to-noise ratio (SNR) for theta-gamma coupling, we considered it the most suitable for a detailed CFD analysis—beyond simply determining its sign—to enable continuous quantification and to validate several model predictions. Prioritizing a high SNR was therefore a key rationale for our choice.

Additionally, given the ongoing debate about the potential contribution of higher harmonics of the underlying theta rhythm to gamma oscillations originating in CA3 (see [25]), we chose to avoid this confound by focusing instead on the lacunosum-moleculare pathway. These considerations led us to prioritize the EC-associated component as a more robust and consistent theta-modulated signal for our analyses.

It is worth noting that in our computational model, we deliberately maintained a general approach by explicitly simulating a single theta-modulated input source. Importantly, our intention was not to model the hippocampus in exhaustive detail, but rather to develop a conceptual model that could aid in interpreting the significance of directionality measures.

Regarding the anatomical specificity of EC inputs to CA1 basket cells, we appreciate the reviewer's precision and have updated the manuscript accordingly. In the revised version, we now cite the study by Bell et al. (2021), which demonstrates that entorhinal afferents indeed form strong, direct glutamatergic synapses onto a substantial fraction of CA1 PV-positive interneurons. In acute mouse hippocampal slices, optogenetic activation of the medial entorhinal alvear pathway (layers II/III)

elicited monosynaptic EPSCs in more than 70% of PV-expressing interneurons, driving them to fire robustly during theta-burst stimulation. Furthermore, selective silencing PV cells reduced entorhinal-evoked feed-forward inhibition of pyramidal neurons by approximately 50%. These findings suggest that EC inputs can recruit CA1 PV interneurons nearly as effectively as the Schaffer collateral pathway.

Finally, the reviewer is correct in noting that references [48, 49] in the previous manuscript focused on the Schaffer collateral pathway and did not provide direct evidence for perforant path activation of basket cells. We apologize for this oversight and have now replaced [48] ([50] in the new manuscript) with the aforementioned study (Bell et al. (2021)). Thank you for highlighting this important point.

[b] Regarding "Cross-frequency coupling and directionality depend on the connectivity": In your models, the initiating driving force of circuit activity is the theta current, which eventually triggers gamma activity. Thus, I would interpret the ground truth (global) directionality as being from theta to gamma in both models. However, the analyses in Figure 1b,c involve CFD computation using local PC signals (namely itransm and VPC), rather than a global network measure. If I understand correctly, in the ING model, basket cells respond more quickly to theta inputs and impose gamma-paced inhibitory currents on PCs, causing gamma to precede theta at the PC soma. In other words, the negative CFD observed for the ING model is exclusively from the point of view of PCs. Although the explanation based on interneuron latency and dendritic integration is plausible, it would be helpful to emphasize more clearly that CFD in this context reflects local temporal precedence rather than external causality. That being said, comparing model-derived CFD (from somatic itransm and VPC) with experimental CFD (based on LFPs) requires further justification. It is not obvious that intracellular somatic signals are equivalent to pathway-specific LFP components in terms of how CFD should be interpreted. Could the authors clarify if I am missing something, or explain why the LFP independent component can be considered analogous to the local PC signals used in the model for interpreting CFD?

<u>Response</u>: *We thank the reviewer for this valuable comment. Indeed, our proposed mechanism is consistent with the reviewer's interpretation: although the external theta input drives the entire network activity, gamma oscillations locally precede theta "recorded" at the PC soma in the ING configuration and occur later in the PING configuration. It is important to emphasize that in actual brain recordings (e.g., LFPs), we do not have direct access to the external input as we do in the model. Instead, the recorded signal reflects the summation of synaptic currents evoked by the input, along with active dendritic currents — the input itself is not directly recorded. Therefore, interpreting CFD from LFP data is more appropriately understood as capturing the local temporal relationship between theta and*

gamma, rather than the directionality between gamma and an unrecorded external driver. In the original manuscript, we attempted to clarify these points (see, for example, lines 79-82 of the Introduction, 329–332 of the Results, and 521–524 of the Discussion), and we have now further revised the text to improve clarity. Thank you.

Although the experimentally recorded LFP is not an exact match for the transmembrane-current or membrane-potential traces generated by our computational model, it does reflect their combined effects—the extracellular summation of excitatory, inhibitory, and active dendritic currents. As such, the model-derived signals provide a close and informative approximation to the LFP, making them well suited for probing theta–gamma interactions. We understand, however, that the reviewer's question relates to whether the measures obtained in the model at the soma level are comparable to those derived from the LFP components, which integrate activity more broadly along the dendro-somatic axis. We agree this is an important consideration. Nevertheless, we believe these measures can serve as a meaningful proxy for investigating CFC and CFD. To address this concern, we have expanded the Results and Discussion sections and included the new Supplementary Fig. S4 showing simulations in which, as in experimental recordings, excitatory and inhibitory inputs arrive at the same compartment, at different somato-dendritic positions. Our results demonstrate that this modification does not alter the directional measure.

We believe it is important to emphasize that our model was not designed to distinguish among the various possible network configurations that could give rise to theta–gamma coupling. In our model, we impose an external theta input and a locally generated gamma rhythm. However, as shown in Supplementary Figure S2 of the original manuscript, the output of our modeled pyramidal cells exhibits clear theta–gamma CFC. Thus, a downstream neuronal layer (not included in our model) receiving this input would display strong CFC at these frequencies, in this case inherited from the upstream activity.

[c] The methods for the experimental analysis presented in Figure 5 are underspecified. It is unclear how gamma frequency was estimated, what signal was used (e.g., an ICA component or raw LFP), and which anatomical layer or gamma band was analyzed. Importantly, previous work from the same group (López-Madrona et al., 2020), based on the same dataset, demonstrated the presence of multiple coexisting gamma oscillations (e.g., slow, mid, and fast gamma) across distinct ICA components, presumably reflecting different afferent pathways; a view consistent with a substantial body of literature on hippocampal gamma oscillations. Based on the frequency bands analyzed here, the authors appear to focus primarily on what the discrete-gamma framework would classify as mid gamma, typically

associated with EC inputs. However, it is also valid to conceptualize mid gamma as an locally generated rhythm, as appears to be the case in the present study, aligning more closely with the continuous-gamma paradigm proposed by Douchamps et al. (2024). If this is indeed the intended framework, it would be helpful for the authors to make this conceptual shift explicit and to provide some justification for adopting it rather than just commenting this quickly in Concluding remarks. Alternatively, the same analysis could be extended to other well-characterized gamma bands to assess generalizability. In any case, these distinctions should be discussed more clearly, as they are critical for interpreting the physiological relevance of the model.

Response: *We appreciate the reviewer's detailed and insightful comments. We have reviewed the caption of Fig. 5 to clarify that the peak in gamma was calculated as the gamma frequency where CFC is maximal. As indicated in the Methods (Dataset) and Results (Cross-frequency coupling and directionality depend on the connectivity) section, the pathway-specific LFP from the stratum lacunosum-moleculare was extracted using ICA and used in the analysis. As the reviewer correctly notes, this component was previously analyzed by our group [12]. In that earlier study, we conceptualized gamma as a locally generated rhythm entrained by pathway-specific inputs hypothesizing that "the CFC reflects a mechanism operated by local excitation-inhibition interactions to coordinate neuronal computations in separate theta frameworks". Furthermore, our results suggested "that local layer-specific circuits interact with upstream afferent pathways to organize hippocampal cell assemblies in multiple theta-gamma frameworks" [12]. Our current modeling approach is consistent with this interpretation. However, for the sake of generality, we implemented a single theta-gamma generating motif, which also aligns with the continuous-gamma framework proposed by [8]. We have now made this connection more explicit in the revised Limitations section. Additionally, we have extended our analysis to explore alternative theta-gamma coupling mechanisms to ensure compatibility with different frameworks on hippocampal oscillatory organization (Suppl. Fig. 4). We trust these clarifications and extensions address the reviewer's concerns.*

[d] The authors refer to their MI analysis as assessing the "encoding capacity" of each model configuration. This terminology is somewhat misleading. As I understand it, the MI (as it is applied here) reflects the extent to which individual pyramidal cell outputs track a specific input signal, essentially quantifying signal fidelity rather than encoding capacity. Thus, high MI in this analysis could result from a trivial scenario in which all PCs reliably and redundantly follow a global input (with a delay). Can we refer to this as high "encoding capacity"? Conventionally, encoding capacity implies

the ability to represent or discriminate between multiple distinct input patterns, to flexibly engage different subsets of PCs depending on the structure of a multidimensional input, or to learn and represent specific population input patterns in the PC outputs so they can be distinguished from each other. Therefore, while the MI analysis used here provides useful insight into the strength of input-output coupling, interpreting it as a direct measure of encoding capacity overstates its significance. It is not clear that MI in this context extracts information beyond what could be obtained using conventional measures such as cross-correlation or coherence. To be clear, I'm not claiming the presented analyses are not insightful; I'm only disputing wording and interpretation. For example, the authors demonstrate that in PING-shifted models, PC output is more reliably modulated by theta input, whereas in ING-shifted models, Poissonian inputs are more effectively reflected in PC activity; the latter likely because ING models do not impose strict gamma-defined windows of opportunity for firing. I welcome clarification from the authors if my interpretations are incorrect.

Response: *We thank the reviewer for this thoughtful and constructive observation. We agree that the term "encoding capacity" may overstate the scope of what is quantified by the mutual information (MI) analysis in our study. As correctly noted, the MI here primarily captures the fidelity with which individual pyramidal cell outputs track a specific input signal, rather than assessing their capacity to represent or discriminate among multiple distinct patterns. To address this, we have revised the manuscript to replace the term "encoding capacity" with "signal fidelity," which more accurately describes the nature of the metric in this context. We believe this change better reflects the interpretive limits of our analysis and avoids potential misunderstandings.*

Minor points

[e] Regarding lines 115-120: If I understand correctly, the authors attribute the difference in gamma frequency between θ-ING and θ-PING primarily to the relatively weak theta input. While I agree that a weak drive likely accentuates the difference by making PC firing sparser in θ-PING, I wonder whether the core reason might lie instead in the architecture of the circuits: in θ-ING, gamma is generated directly by fast-spiking BCs under theta drive, whereas in θ-PING it depends on the slower recruitment of BCs via pyramidal cell firing. Perhaps the authors could clarify whether this structural distinction, rather than input strength per se, is the main determinant of the frequency difference, or explain why that interpretation would be inaccurate.

Response: *We do not attribute the difference in the γ-frequency between θ-ING and θ-PING to the strength of the θ drive. As the reviewer correctly suggests, the decisive factor is the circuitry architecture. In the θ-ING configuration, θ-modulated excitation directly targets BCs whose fast intrinsic properties and rapid* $GABA_A$ *synapses allow them to fire at a high-γ rhythm (Fig. 1b). In contrast, in the θ-PING configuration, the same θ input must first depolarise pyramidal cells, whose slower membrane time constants and glutamatergic synapses effectively low-pass-filter the drive. Only then do the BCs become recruited via the additional PC→BC step, resulting in inherently slower γ oscillation (Fig. 1c). We have clarified this mechanism in the revised Model section to avoid any confusion. We appreciate the opportunity to make this point more explicit.*

[f] Regarding Fig. 3: I understand that the instantaneous firing rates shown in panels (v) are normalized to facilitate visualization of timing differences. However, it would help readers more fully grasp the results if some indication of the relative peak firing rates across conditions were also provided. For example, showing how much the overall firing rate of the pyramidal cell in the blue trace is reduced compared to that in the orange trace.

Response: *We appreciate the reviewer's suggestion to improve the clarity regarding differences in firing rate magnitudes across conditions. As noted in Fig. 3, we included colored arrows in panels (v) to qualitatively illustrate whether firing rates increase or decrease with changes in the corresponding synaptic weights. In addition, the slope of these traces provides a qualitative indication of rate differences.*

For readers interested in precise numerical comparisons, we have provided detailed firing rate values in Supplementary Table S4, allowing for a clear, quantitative assessment of peak firing rates across conditions without overcrowding the main figures.

Reviewer #2: This manuscript integrates neural circuit dynamics modeling with experimental data analysis to explore how feedforward and feedback inhibition regulate the directionality of cross-frequency coupling in the theta-gamma band. The study reveals the mechanistic origins of cross-frequency directionality under different connections, and further validates its modeling predictions by incorporating in vivo electrophysiological data from the rat hippocampus. Overall, this research holds high scientific value and publication potential, though several minor revisions need to be addressed:

1. What do the different colors in the figures represent? It is recommended to add color legends or brief annotations for clarity.

<u>Response</u>: *We thank the reviewer for the positive assessment of our work. Regarding the figures, we have aimed to keep them as self-explanatory as possible. Wherever space permitted, legends or on-plot labels identify each trace or symbol. In the case of dense raster plots—specifically in Fig. 1b-ii, 1c-ii, 2a, and 4b—inserting legends directly within the panels would obscure the spike trains. To address this, the figure captions explicitly describe the color codes and visual conventions used.*

We have carefully reviewed all figure captions to ensure that the color meanings are clearly described and consistent across the manuscript. We believe this approach preserves the readability of the figures while providing unambiguous guidance to the reader.

2. Can similar gamma-to-theta phenomena be observed if the external theta input contains noise? The model should consider robustness under noisy conditions.

<u>Response</u>: *We thank the reviewer for raising this important point. Our model does, in fact, incorporate noise in the external theta input. Specifically, the interval between consecutive theta peaks,* T_{cycle}*, was drawn from a Gaussian distribution with a mean of* $\mu_T = 125$ *ms and a standard deviation of* $\sigma_T = 16$ *ms. Within each cycle, 10,000 spikes were distributed according to a second Gaussian with a standard deviation of* $w = 25$ *ms. This introduces temporal variability that effectively mimics a noisy theta input (see figure below).*

To further clarify this aspect, we have prepared an illustrative figure (included below for the review process only, unless its inclusion is required in the final version) demonstrating the variability of the theta input and its impact on the model's behavior. We believe this adequately addresses concerns about the robustness of gamma-to-theta phenomena under noisy conditions.

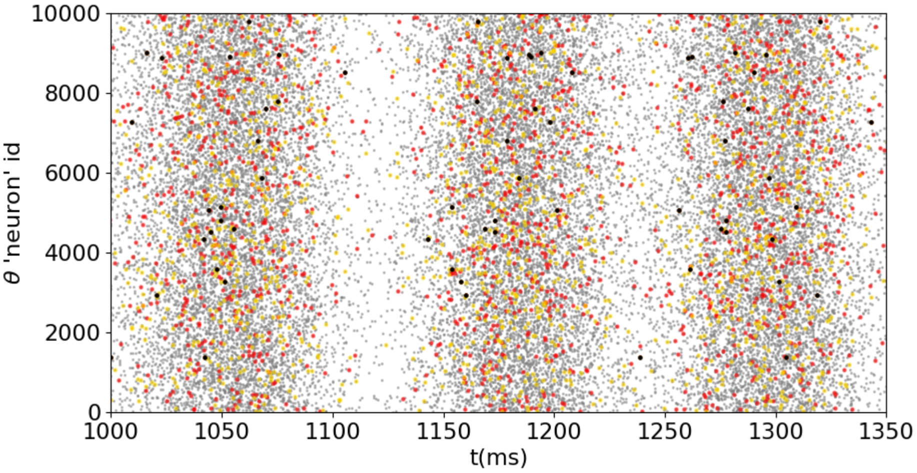

Figure Caption: *Spikes originating from the theta input population arriving at the PCs and BCs. The entorhinal cortex input originates from 10.000 "virtual neurons", each producing one spike per theta cycle. Spikes arriving at CA1 at two randomly chosen target neurons are shown in red and yellow, while spikes delivered to both targets simultaneously are marked in black. Other spikes not arriving at the target neurons are shown in gray and rendered smaller for clarity. The figure illustrates the high level of variability introduced in the theta input.*

3. The model includes parallel pathways, while the experiments focus on a single channel. If the model is hypothetical, its assumptions should be further clarified, and the rationality of the model structure should be explained.

Response: *We appreciate the reviewer's comment. Our model is intentionally minimal, designed to capture a canonical circuit motif relevant across multiple regions. Specifically, in the case of hippocampal area CA1—which is the focus of the experimental data presented in Fig. 5—there is clear anatomical and physiological evidence for convergent input from two primary pathways: CA3 via the Schaffer collaterals and layer III of the EC via the temporoammonic pathway. In our model, these correspond to two distinct pathways: the CA3 is modelled through the parallel pathway, and the EC input is modelled through the external theta-modulated drive.*
We have aimed to clarify this rationale in the Discussion section (Experimental Evidence and Interpretation of CFD Measurements) and to specify the key model assumptions, including simplified circuit elements and connectivity, in the Methods section. In response to this comment, we have further revised the Methods to more explicitly explain the model's structure, its justification, and its scope of applicability.

4. The experimental sample size is relatively small (5 rats). It is suggested to discuss limitations in the manuscript and propose hypotheses for generalizing the findings to other brain regions or non-human primates (e.g., monkeys).

Response: *Thank you for this comment. It is important to note, that a sample size of five animals, combined with multiple longitudinal recordings across different days and sessions, is well within the standards of the field and provides robust, reproducible data, even though a larger sample could naturally strengthen the conclusions further. Crucially, the dataset analyzed here is the same one that previously demonstrated distinct theta–gamma frameworks in the hippocampus with negative CFD—a central observation that directly motivated the present computational work. Using this established dataset to validate our model predictions is scientifically sound and appropriate.*

In addition, our Discussions address the potential generalization of our findings to other brain regions, including the neocortex, and in humans [30]. To maintain focus and avoid overextending the interpretation, we believe the current version of the Experimental Evidence and Interpretation of CFD Measurements section sufficiently discusses these aspects while remaining balanced and concise.

5. The calculation logic and intuitive meanings of CFD and CFC need more detailed explanation. These should be elaborated in the Methods section or Supplementary Materials. Additionally, since there is no illustration showing what "positive and negative values" represent in terms of temporal precedence, it is recommended to include a schematic diagram in the main text or Figure 1 to clarify that positive and negative CFD values correspond to "theta leads gamma" and "gamma leads theta," respectively.

Response: *In response to the reviewer's comment, we have added the new Supplemental Figure 1 to illustrate the difference between coupling and directionality, including a schematic diagram that explicitly clarifies the interpretation of positive and negative CFD values correspond to temporal precedence—that is, "theta leads gamma" versus "gamma leads theta".*

6. Regarding computational modeling: As the study uses a simplified model, the simplification conditions and rationality of parameter selection need to be addressed—for example, through parameter sensitivity analysis and explicit justification of parameter choices.

Response: *We thank the reviewer for highlighting this aspect. Our primary goal was to develop a model that balances simplicity with the ability to reproduce key features of cross-frequency coupling (CFC), ensuring its relevance and applicability across experimental and theoretical contexts. To achieve this,*

we relied on well-established neuron models for PCs and BCs drawn from prior literature [32], providing biological plausibility while maintaining computational tractability.

The network size was scaled down to allow for efficient simulation while preserving essential properties, such as a realistic excitatory-to-inhibitory cell ratio. As a result of this downscaling, synaptic weights were not directly matched to experimental values but were tuned so that firing rates fell within empirically observed ranges (below 5 Hz for PCs and well above 5 Hz for BCs [22]).

Although we did not conduct a formal sensitivity analysis, we systematically explored parameter ranges and performed extensive adjustments during model development. These tests showed that the main qualitative results—particularly the emergence and directionality of CFC—were robust across a broad range of parameter values, except in extreme conditions. This robustness is explicitly demonstrated in Figures 3 and 4, where we note that "CFD in the θ-ING model remains negative across a broad range of synaptic weights (see Table S3) and PC activity (see firing rates in Table S4)." Notable transitions in directionality only occurred under specific conditions, such as excessively strong inhibition to PCs (Figs. 3a-v and 3c-i) or low external excitation to BCs (Fig. 3c-i).

In response to this comment, we have added further details to the Methods section to clarify parameter selection and assumptions, and we expanded the Results and Discussion to include new analyses shown in Supplementary Figure S4, demonstrating that manipulations of θ-ING connectivity do not alter the directionality outcome. We believe these additions address the reviewer's concerns thoroughly.

Reviewer #3: In this manuscript, the authors study theta-gamma cross-frequency coupling as a function of the structure of neuronal circuits. Namely, they show that feed-forward inhibition exhibits dominant gamma to theta interactions, whereas there as the feedback inhibition mediated the reverse interactions. In addition, the authors also study the mixed circuit where they investigate different strengths of theta-BC drive and PV-BC coupling. They compared their results with the experimental data where rats explored familiar or novel environments.

This is a very interesting, well done study, with important implications on how the hippocampal circuit may function at different stages of information processing.

<u>Response</u>: *We thank the reviewer for the positive assessment.*

I have however few questions/comments that I would like the authors to answer:

1. the theta drive in the hippocampus is known to come from common source - medial septum. The authors implement dendritic delay on BC cells but not PC cells. why? Is it because PC compartments induce intrinsic delay? Where is the value of this delay taken from?

<u>Response</u>: *We appreciate the reviewer's observation. In our model, PCs are implemented with a multi-compartmental structure that captures the spatial and temporal dynamics of dendritic integration, including intrinsic propagation delays arising from electrotonic distance and active channel dynamics. As a result, it is not necessary to impose an explicit dendritic delay on PCs, since these delays naturally emerge from the compartmental architecture.*

To validate this, we compared the propagation of synaptic inputs arriving at the distal dendrites versus those delivered directly to the soma, and observed a transmission delay of approximately 3 ms in our model. This value is consistent with experimental reports, which indicate that dendritic signals in PCs can reach the soma in about 2 ms when triggering dendritic spikes ([43] in the new manuscript), and up to 5 ms for passively propagated EPSPs ([42] in the new manuscript). We have updated the methods section and the references so that our modeling choices are better clarified.

2. The conclusion drawn from experimental data seems a bit overdrawn. Based on Figure 3 similar results to those observed experimentally could be obtained just by strengthening BC->PC connection in the ING circuit, rather than introducing the PING circuit. This should be discussed.

<u>Response</u>: *We thank the reviewer for highlighting this point. While strengthening the BC→PC synapse within a pure θ-ING circuit can indeed influence directionality, this adjustment alone does not account for the observed shift in peak of gamma (see Fig. 3a). Critically, the experimental data presented in Fig. 5 demonstrate that increases in CFD are accompanied by a decrease in gamma frequency. Moreover, the known anatomical and physiological connectivity of CA1 supports a mixed θ-ING/θ-PING architecture, consistent with our model results showing that increased gamma frequency is associated with more negative CFD values (see Fig. 4) and the associated Results session.*

3. There is experimental data showing that PV+ population mediates theta rhythm in hippocampus, and at the same is critical in consolidating newly acquired memory (see for example Ognjanovski et al, Nat. Comm 2017). What is experimental evidence for not including the theta drive to PV+ cells in the PING model?

Response: *We thank the reviewer for this comment and for highlighting relevant experimental evidence. We fully acknowledge that PV+ interneurons are key contributors to theta rhythms in the hippocampus and play a critical role in memory consolidation. Our initial analysis deliberately separated ING and PING mechanisms to clarify their contributions to cross-frequency dynamics in a controlled, systematic manner.*

However, we agree that this separation represents a simplification. In Figures 4 and 6, we present a more integrated model in which both ING- and PING-related connections coexist, providing a more physiologically realistic scenario that aligns with experimental findings on PV+ cell involvement. The advantage of the computational approach is precisely the ability to dissect specific circuit motifs in isolation, which is not feasible experimentally, thereby allowing us to better understand their distinct roles before re-integrating them.

4. Could authors explain why do changes in CFD organize vertically in the ING model (i.e are gamma frequency dependent; similarly to the data) whereas they organize horizontally in the PING model (i.e. are theta frequency dependent)?

Response: *We thank the reviewer for this insightful question regarding the distinct CFD patterns between the θ-ING and θ-PING motifs. In Fig. 4, we present systematically how CFD changes across mixed and pure θ-ING/θ-PING configurations. A clear pattern emerges along the* f_{ampl} *axis, where positive CFD values cluster at lower gamma frequencies and negative values dominate at higher gamma frequencies. This vertical organization reflects a fundamental aspect of our framework: higher-frequency gamma oscillations are characteristic of θ-ING dynamics, whereas slower gamma rhythms are more consistent with θ-PING activity. Notably, this same pattern, with negative CFD at higher gamma frequencies, is also observed in the experimental data (Fig. 1a-iii).*

We acknowledge, however, that in the θ-PING motif (e.g., Fig. 1c-vi), the CFD pattern may be influenced by intrinsic limitations of the metric itself. Gamma cycles generated in θ-PING models tend to have higher amplitudes than in θ-ING, producing sharper events that, in time-frequency decomposition, spread across multiple frequency components. This could explain the horizontal organization (theta-frequency dependence) seen in CFD for θ-PING.

It is important to emphasize that CFD is reliable only in regions of significant CFC, where a meaningful phase difference between theta and gamma can be estimated. For this reason, when we compute the predominant CFD_{avg}*, we include only CFD values within areas of high CFC (see Methods section).*

Outside these regions, the phase-slope-based CFD measure can become less reliable and susceptible to factors such as the distribution of gamma activity across the theta cycle (e.g., broad versus narrow phase locking) or distortions due to non-sinusoidal theta waveforms, which can bias phase estimation.
